# Failure of senolytic treatment to prevent cognitive decline in a female rodent model of aging

**DOI:** 10.3389/fnagi.2024.1384554

**Published:** 2024-05-15

**Authors:** Asha Rani, Linda Bean, Vivekananda Budamagunta, Ashok Kumar, Thomas C. Foster

**Affiliations:** ^1^Department of Neuroscience, McKnight Brain Institute, University of Florida, Gainesville, FL, United States; ^2^Genetics and Genomics Graduate Program, Genetics Institute, University of Florida, Gainesville, FL, United States; ^3^Department of Pharmacodynamics, College of Pharmacy, University of Florida, Gainesville, FL, United States

**Keywords:** aging, hippocampus, spatial memory, senolytic, cognitive testing

## Abstract

There are sex differences in vulnerability and resilience to the stressors of aging and subsequent age-related cognitive decline. Cellular senescence occurs as a response to damaging or stress-inducing stimuli. The response includes a state of irreversible growth arrest, the development of a senescence-associated secretory phenotype, and the release of pro-inflammatory cytokines associated with aging and age-related diseases. Senolytics are compounds designed to eliminate senescent cells. Our recent work indicates that senolytic treatment preserves cognitive function in aging male F344 rats. The current study examined the effect of senolytic treatment on cognitive function in aging female rats. Female F344 rats (12 months) were treated with dasatinib (1.2 mg/kg) + quercetin (12 mg/kg) or ABT-263 (12 mg/kg) or vehicle for 7 months. Examination of the estrus cycle indicated that females had undergone estropause during treatment. Senolytic treatment may have increased sex differences in behavioral stress responsivity, particularly for the initial training on the cued version of the watermaze. However, pre-training on the cue task reduced stress responsivity for subsequent spatial training and all groups learned the spatial discrimination. In contrast to preserved memory observed in senolytic-treated males, all older females exhibited impaired episodic memory relative to young (6-month) females. We suggest that the senolytic treatment may not have been able to compensate for the loss of estradiol, which can act on aging mechanisms for anxiety and memory independent of cellular senescence.

## 1 Introduction

There are sex differences in vulnerability and resilience to the stressors of aging and the incidence or severity of age-related cognitive decline. Episodic memory begins to exhibit a marked decline around middle age in humans (Nyberg and Pudas, 2019) and rodent models (Foster, 2012; Febo et al., 2020). During middle age, women outperform men on tasks of episodic memory and women exhibit a superior trajectory of cognitive decline relative to men (Lundervold et al., 2014; McCarrey et al., 2016; Olaya et al., 2017; Casaletto et al., 2019). The trajectory of cognitive decline and a decrease in hippocampal volume during aging is associated with increased levels of markers for systemic inflammation. Moreover, this relationship is stronger for males relative to females (Jefferson et al., 2007; Schmidt et al., 2016; Walker et al., 2019), suggesting a greater influence of systemic inflammation in males. As such, sex differences in the immune system may determine the effectiveness of treatments designed to preserve cognitive function.

Cellular senescence, one of the hallmarks of aging, occurs in response to damaging or stress-inducing stimuli. The response includes a state of growth arrest and the development of a senescence-associated secretory phenotype (SASP), with the release of several pro-inflammatory cytokines and chemokines that contribute to the rise of systemic inflammation during aging (Baker et al., 2011; Kirkland and Tchkonia, 2020; Budamagunta et al., 2023). Pro-inflammatory molecules, released from senescent cells in the periphery, induce oxidative stress and inflammation in the brain, and stress-responsive signaling of brain cells (Budamagunta et al., 2023). The resulting change in neurophysiology can be considered a compensatory response to limit intracellular Ca^2+^ and excitotoxicity; however, the gain in neuroprotection is at the expense of function, resulting in cognitive impairment (Foster, 1999; Kumar and Foster, 2007; Kumar et al., 2009). Senolytics are a class of drugs designed to selectively eliminate senescent cells from the body and potentially delay or alleviate age-related disorders. Our previous work employed two different senolytic treatments, a dasatinib + quercetin cocktail (D + Q) or ABT-263. The two treatments act on different mechanisms to promote apoptosis of senescent cells (Chan et al., 2012; Zhu et al., 2016) and quercetin is an antioxidant (Ishisaka et al., 2014). Importantly, dasatinib and quercetin, but not ABT-263, cross the blood brain barrier (Porkka et al., 2008; Yamaguchi and Perkins, 2012; Ishisaka et al., 2014; Mehdipour et al., 2021; Budamagunta et al., 2023, 2024). Both treatments improve cognition in males, emphasizing a link between cognitive decline and senescent cells in the periphery (Budamagunta et al., 2023, 2024). However, there are several mechanisms involved in aging and sex differences, particularly hormonal differences, contribute to age-related cognitive decline (Foster, 2005; Bean et al., 2014). Therefore, the current pilot study addresses the question of whether senolytic treatment can maintain cognition in aging females over the time in which estrogen levels normally decline. The results demonstrate that senolytic treatments did not yield evidence of preserving memory in female rats and may have increased sex differences in behavioral stress responsivity.

## 2 Experimental procedures

### 2.1 Animals

Procedures and experiments pertaining to animals have been reviewed and approved by the Institutional Animal Care and Use Committee (IACUC) of University of Florida. All the procedures and experiments involving animals were in accordance with the guidelines set forth by the United States Public Health Service Policy on Humane Care and Use of Laboratory Animals. This study utilized young (6 months) and middle-aged (12 months) female Fischer 344 rats obtained from the National Institute on Aging through University of Florida animal care services. The animals were maintained in a reverse cycle 12:12 h light/dark schedule. Food and water were provided *ad libitum*. The ages, treatments, and behavioral testing schedule were designed to mimic, as closely as possible, our previous work examining senolytic treatment in male Fischer 344 rats (Budamagunta et al., 2023).

### 2.2 Treatments

**FIGURE 1:**
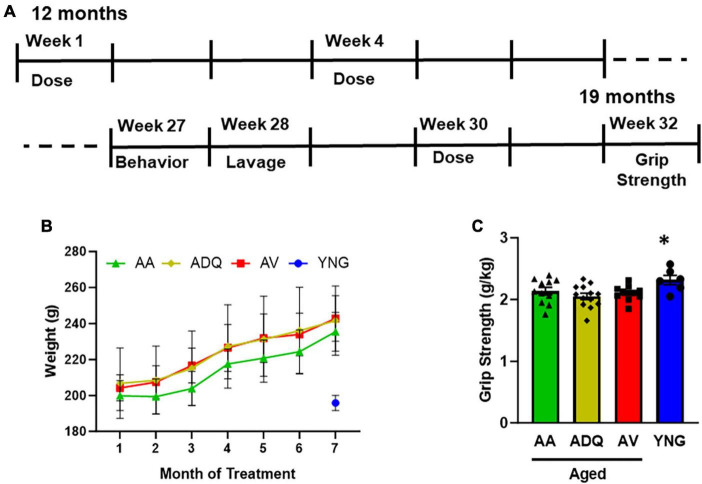
No effect of treatment on weight and grip strength. **(A)** Schematic of the time course of treatment and testing. Rats were treated *via* oral gavage for 5 consecutive days with a 2 week break in between treatment cycles. Animals were behaviorally tested during week 25 followed by a final treatment on week 27. Final weight and grip strength were measured on week 30, 2 weeks after the final treatment. **(B)** Mean weight (±SEM) for older animals over the course of treatment and weight of the Young (YNG) group (blue circle) measured at the same time as the final weight for aged animals. **(C)** Grip strength normalized to weight. The asterisk indicates significantly greater grip strength of YNG relative to the three older groups.

Rats (total = 41) were allowed to acclimatize to their new animal facility and the reverse light cycle schedule for at least 10 days before the initiation of any procedure. Middle age rats (12 months; total = 35) were divided randomly into three groups of which one group received a vehicle treatment (AV, *n* = 11), another group received a dasatinib (1.2 mg/kg) + quercetin (12 mg/kg) cocktail (ADQ, *n* = 13), while the final group received ABT-263 (12 mg/kg) treatment (AA, *n* = 11). The dose of dasatinib + quercetin was based on our previous work examining cognition and peripheral markers (Budamagunta et al., 2023). The drugs were dissolved in a vehicle containing 60:30:10 ratio of Phosal 50 PG, PEG400 and ethanol, respectively. Rats were treated *via* oral gavage for 5 consecutive days followed by a 2 week break in between treatment cycles (Figure 1A). A total of 10 cycles of treatment over the span of 7 months were administered before the rats were behaviorally characterized. A final round of senolytic treatment was administered a week after the completion of the behavioral characterization and rats were euthanized 2 weeks following the final treatment. Young animals (6 months) (YNG; *n* = 6) were tested at the same time as older groups.

Hormonal status of females contributes to variability in episodic memory on the watermaze and an age-related decline in episodic memory on the watermaze is linked to the decline in estradiol (Warren and Juraska, 2000; Bean et al., 2014; Rentz et al., 2017). Furthermore, there may be a relationship between hormone levels and cell senescence which influences the effectiveness of treatments designed to preserve cognitive function (Casaletto et al., 2020; Polcz et al., 2023). Therefore, prior to treatment, all 12-month animals were examined for estrus cycle. Vaginal lavage was performed for 11 consecutive days between 9:00 and 11:00 a.m. Vaginal cells were collected from each animal using a smooth-tipped glass eye dropper and 1 drop (∼20 μl) of sterile 0.9% saline. Vaginal cells were placed on glass slides and stained with cresyl violet. The phase of the estrous cycle was recorded after viewing the slide under low magnification on a light microscope. Determination of the estrous phase (proestrus, estrus, metestrus, and diestrus) was based on the cytology of the collected cells (nucleated epithelial cells, cornified squamous epithelial cells, and leukocytes) according to previous reports (Marcondes et al., 2002; Bean et al., 2015). No cycle assessments were conducted during the treatment period in order to minimize differential handling relative to our previous work in aging males (Budamagunta et al., 2023). For a subset of older animals (*n* = 3 per group), the cycle was examined for 5 consecutive days following testing on the watermaze and prior to the final treatment.

### 2.3 Behavior

#### 2.3.1 Cue discrimination task

Animals were tested on the cue discrimination and the 1-day version of the spatial watermaze task, starting 1 week following 8 cycles of treatment (∼6 months after initial treatment). Details of behavior measures have been previously described (Guidi et al., 2014; Smith et al., 2020; Barter et al., 2021; Budamagunta et al., 2023). A black circular (1.7 m diameter) water tank, located within a well-lit room was surrounded by a black curtain. The temperature of the water was maintained at 27 ± 2°C. An escape platform, roughly 1 cm above the water level, held a white visual cue. Noldus EthoVision software was used to record and process data from the trials. Before testing, rats were separated into individual cages. After 20 min of acclimatization to the new cages, the animals were habituated to the pool by letting them swim freely for 30 s. Behavioral training consisted of five training blocks of three trials each and the entirety of cue discrimination training was completed in 1 day. The inter-trial interval was 20 s and inter-block interval was 20 min. At the end of each block, the animal was returned to its cage, which was placed in front of a heater to prevent hypothermia. Release points, platform, and start locations were randomized for each trial. Rats were given 60 s per trial to find the platform and if they failed to do so, they were gently guided to the platform.

#### 2.3.2 Spatial discrimination task

Three days after the cue discrimination training, animals were trained on the 1-day spatial version of the watermaze to assess their ability to use the distally placed spatial cues to remember and navigate to the location of the submerged platform (Foster and Kumar, 2007; Guidi et al., 2014, 2015; Kumar et al., 2018; Barter et al., 2020, 2021; Budamagunta et al., 2023). Bright and contrasting objects were placed on all four sides of the pool to act as distally located spatial cues. The escape platform was submerged 1 cm below the water surface and the platform location was fixed throughout the duration of the spatial discrimination training. The training consisted of five blocks of three trials per block and the start location for each trial was changed randomly for each trial. Each rat was given 60 s to find the location of the platform and if they failed to find the platform within the 60 s, they were gently guided to the platform. The inter-trial interval was 20 s and the inter-block interval was 20 min. At the end of each block, the rat was returned to the holding cage, which was placed next to a heater to prevent hypothermia.

At the end of the fifth block, an acquisition probe trial was performed. The platform was removed from the pool and each rat was released from the quadrant opposite the goal quadrant, where the platform was initially located. During the probe trial, the rat was allowed to swim freely for 60 s. After the end of the acquisition probe trial, a refresher block of training with the platform placed back into the goal quadrant was administered. 24 h after spatial training, the rat was tested on the retention probe trial where the platform was again removed from the pool and the rat was allowed to swim for 60 s. To quantitatively assess the performance on the probe trials, discrimination index (DI) scores were calculated using the formula [(time spent in goal quadrant − time spent in opposite quadrant) / (time spent in goal quadrant + time spent in opposite quadrant)].

#### 2.3.3 Inhibitory avoidance

One week following spatial watermaze training, an inhibitory avoidance test was conducted based on the protocols established previously (Foster et al., 2003; Foster and Kumar, 2007; Zhou et al., 2011; Speisman et al., 2013; Budamagunta et al., 2023). In short, an inhibitory avoidance apparatus (Coulbourn Instruments, Allentown, PA, USA) comprising two compartments connected by an automatic door was used for this test. One of the chambers was lit by a light, while the other chamber was maintained dark. On the training day, one rat at a time was put into the light chamber and was allowed to acclimatize for 90 s. The connecting door was programed to automatically open at 90 s, allowing the rat to access the dark chamber. The rat was given 10 min to enter the dark chamber and once all four paws of the rat crossed over to the dark chamber, the automatic door was shut and the rat was given a relatively mild electric shock (0.21 mA) for 3 s. This usually elicits a rapid movement response in the rat, which confirms the rat received an electric shock. Five seconds later, the rat was removed from the chamber and returned to its home cage.

On the testing day (24 h after the training trial), the rat was once again placed in the light chamber and allowed to acclimatize for 90 s before the connecting door opened. The rat was then given 10 min to re-enter the dark chamber at the end of which the rat was returned to their home cage. The latency to re-enter the dark chamber was recorded and used to assess memory.

#### 2.3.4 Grip strength test

Grip strength was determined as described previously (Carter et al., 2009; Cui et al., 2009; Zhou et al., 2011; Kumar et al., 2012; Budamagunta et al., 2023). Briefly, grip strength was assessed using an automated grip strength meter by sensing the peak amount of force an animal applies in grasping the pull bar assembly (Columbus Instruments, Columbus, OH, USA). The rat was hand-held by the experimenter using assembly (Columbus Instruments, Columbus, OH, USA). For each measurement, the rat’s forelimbs were gently placed on the bar, the animal grabbed the bar (a reflex response in rodents) and was then drawn along a straight line leading away from the sensor. The rat released the pull bar at some point and the maximum force attained was stored on the digital display. The peak amount of force the animal applied in grasping the pull bar was measured. The mean force (grams) was calculated over four trials and was divided by body weight. The mean force (grams) was calculated over three trials, separated by 2–4 min.

## 3 Results

### 3.1 Behavior and cognitive function

The treatment and testing procedures are similar to our previous work examining the effect of senolytic treatments on cognition in male rats (Budamagunta et al., 2023). Middle age female F-344 rats (12 months) were randomly divided into three groups that received a vehicle treatment (AV, *n* = 11), a dasatinib (1.2 mg/kg) + quercetin (12 mg/kg) cocktail treatment (ADQ, *n* = 13), or ABT-263 (12 mg/kg) treatment (AA, *n* = 11) over the course of aging (7 months of treatment). Young animals (6 months) (YNG; *n* = 6) were tested at the same time as the older groups for grip strength, cue discrimination, episodic spatial memory on watermaze, and inhibitory avoidance.

#### 3.1.1 Weight and grip strength testing

For the older groups, weight was examined during the 7 months of treatment (Figure 1B) and the final weight was compared to that of the YNG. A repeated measures ANOVA indicated that the older groups increased weight over the course of treatment [*F*(6,192) = 346.87; *p* < 0.0001] in the absence of a treatment effect. The increase in body weight with age is similar to previous reports for female F344 rats (Turturro et al., 1999). An ANOVA for the final weight indicated a difference across groups [*F*(3,37) = 22.24; *p* < 0.0001] due to increased weight of all older groups AV, AA, and ADQ relative to YNG (Figure 1B). Grip strength, normalized to weight, exhibited a group difference [*F*(3,37) = 3.26; *p* < 0.05]. *Post-hoc* test indicated that grip strength decreased in all older groups relative to YNG (Figure 1C).

#### 3.1.2 Cue discrimination training

**FIGURE 2:**
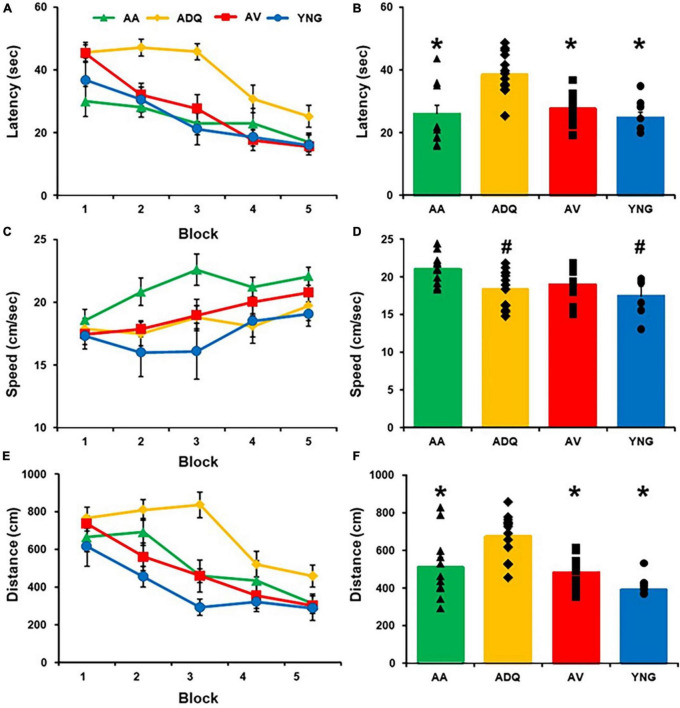
Effect of senolytic treatments on cue discrimination behavior. Mean (±SEM) latency **(A)**, swim speed **(C)**, and escape distance **(E)** for each training block for the cue discrimination task. The mean (+SEM) latency **(B)**, swim speed **(D)**, and escape distance **(F)** averaged across blocks illustrates that the ADQ group exhibited an increase in latency and distance to escape and the AA group exhibited an increase in swim speed on the cue task. Asterisks indicate a significant difference relative to the ADQ group. Pound signs indicate a significant difference relative to the AA group.

A repeated measures ANOVA of the latency to reach the platform across training blocks for the cue discrimination task revealed a significant effect of training [*F*(4,148) = 26.92; *p* < 0.0001], a group difference [*F*(3,37) = 10.54; *p* < 0.0001], and an interaction [*F*(12,148) = 1.93; *p* < 0.05] (Figure 2A). *Post hoc* analyses indicated that each group exhibited a decrease in latency across training (*p* < 0.05) and the ADQ group had an increased latency relative to all other groups (Figure 2B). Swim speed increased with training [*F*(4,148) = 6.65; *p* < 0.0001] and exhibited a group difference [*F*(3,37) = 4.25; *p* < 0.05], in the absence of an interaction (Figure 2C). *Post hoc* comparisons indicated the increase in swim speed across blocks of training (*p* < 0.05) was limited to the AA and AV groups and the AA group had a greater swim speed relative to the other three groups (Figure 2D). A repeated measures ANOVA on the distance to reach the platform across training trials for the cue discrimination task revealed a significant effect of training [*F*(4,148) = 22.49; *p* < 0.0001] and a group difference [*F*(3,37) = 8.65; *p* < 0.0005], in the absence of an interaction (Figure 2E). *Post hoc* analyses indicated that each group exhibited a decrease in distance across training (*p* < 0.05) and the ADQ group had an increased distance relative to all other groups (Figure 2F). Thus, all groups exhibited a decreased latency and pathlength with training, indicating learning of the cue discrimination, although differences due to treatment were noted with the longer latency and pathlength for ADQ group and faster swim speed for AA animals.

#### 3.1.3 Spatial discrimination training

**FIGURE 3:**
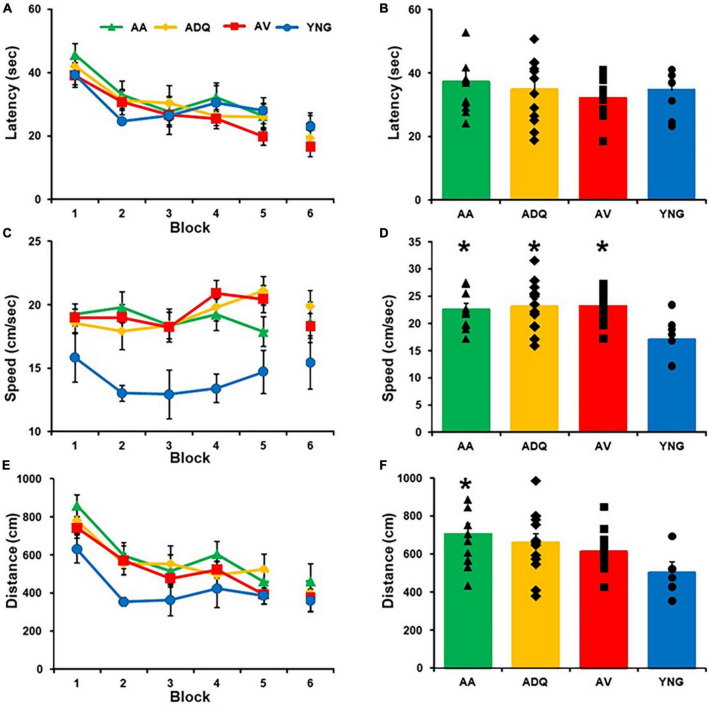
Effect of senolytic treatments on spatial discrimination behavior. Mean (±SEM) latency **(A)**, swim speed **(C)**, and escape distance **(E)** for each training block for the spatial discrimination task. The mean (+SEM) latency **(B)**, swim speed **(D)**, and escape distance **(F)** averaged across blocks illustrate that the YNG animals exhibited a slower swim speed and shorter distance, with no differences between all the older groups. Asterisks indicate a significant difference relative to the YNG group.

Three days following cue discrimination training, animals were tested on the 1-day version of the spatial watermaze, which is sensitive to age-related impairment of episodic spatial memory (Foster et al., 2003; Foster, 2012; Bean et al., 2015). A repeated measures ANOVA across training blocks, on the latency of rats to reach the platform yielded a significant effect of training [*F*(5,185) = 16.86; *p* < 0.0001] (Figure 3A) in the absence of a treatment effect or an interaction (Figure 3B). A tendency (*p* = 0.052) for group difference in swim speed (Figure 3C) was due to slower swim speed of YNG, relative to the three older groups (Figure 3D). A repeated measures ANOVA performed across training blocks, on the pathlength to escape (distance) yielded a significant effect of training [*F*(5,185) = 15.70; *p* < 0.0001] and a tendency (*p* = 0.097) for a group effect (Figure 3E). A *post hoc* analysis of the average distance across all blocks indicated that AA swam a greater distance relative to YNG (Figure 3F). However, no group differences were observed for any single block of training.

**FIGURE 4:**
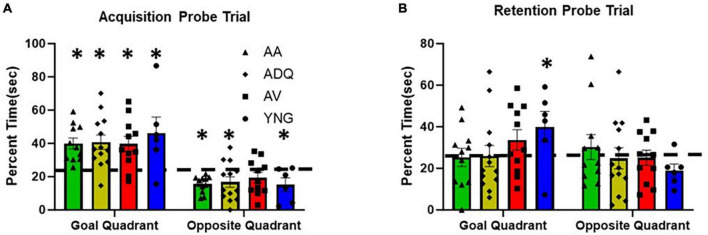
No effect of senolytic treatments on spatial memory. The figure provides the percent time spent in the goal and opposite quadrants (mean + SEM) for the **(A)** acquisition and **(B)** retention probe trials on the watermaze. Asterisks indicate a difference relative to chance (25%).

No group difference was observed for the percent time searching the goal quadrant during the acquisition probe trial and one-group *t*-tests indicated that all groups performed above chance (*p* < 0.05) (Figure 4A). Similarly, no group differences were observed for percent time in the opposite quadrant and all groups except AV were below chance. Examination of the 24-h retention probe trial indicated no group differences in percent of time searching the goal quadrant or the opposite quadrant. However, only YNG were above chance, and AV exhibited a tendency (*p* = 0.053) to increase searching of the goal quadrant during retention testing (Figure 4B). Finally, YNG tended to be below chance (*p* = 0.053) in searching the opposite quadrant during retention testing. The results confirm an age-related impairment in memory and indicate no benefit from senolytic treatments.

#### 3.1.4 Inhibitory avoidance

**FIGURE 5:**
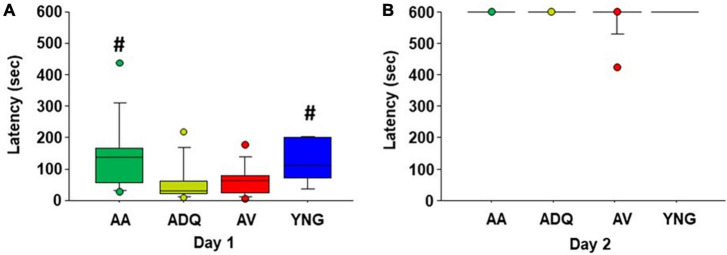
No effect of age or senolytic treatments on memory for inhibitory avoidance. Box plots for latency to enter the dark compartment of inhibitory avoidance during **(A)** day 1 training and **(B)** day 2 retention testing. Pound signs indicate a difference in latency on day 1 relative to AV and ADQ.

One week following spatial watermaze training, an inhibitory avoidance test was conducted. A Kruskal–Wallis one-way ANOVA indicated a group difference (*H* = 10.53, *p* < 0.05) in the latency to cross into the dark chamber on the training day (day 1). *Post hoc* Mann–Whitney U tests indicate that the latency to enter the dark compartment was increased for AA and YNG relative to ADQ and AV (Figure 5A). Kruskal–Wallis test for latency to the dark chamber on the retention testing day was not significant (Figure 5B).

#### 3.1.5 Reproductive status

All middle age animals were examined by vaginal lavage for at least 2 cycles (11 consecutive days) and all were exhibiting a regular estrus cycle (5.29 ± 0.22 days) prior to treatment. At 19-months of age, there was not a discernible estrous cycle pattern detected in either drug treatment groups, or in the vehicle treated group (*n* = 3 per group). Animals were therefore determined to be acyclic.

## 4 Discussion

Comparison of the current results with our previous work in males reveals several sex differences. Foremost was the marked beneficial effects of senolytic treatment in preserving grip strength and cognitive function in aging male rats (Budamagunta et al., 2023). In contrast, similar senolytic treatments in female rats did not provide evidence for improved grip strength or memory on the watermaze. The older animals experienced more handling due to the treatment procedures suggesting some sex differences may relate to anxiety/stress associated with different behavioral context including the level of handling and exposure to the water maze (Bohacek et al., 2015; Sensini et al., 2020; Eliot et al., 2023). The effect of age on swim speed was different for males and females. For males, swim speed on the cue and spatial task decreased with age and was not altered by the senolytic treatments (Budamagunta et al., 2023). In the current study, all older female groups, including vehicle treated, exhibited an increase in swim speed relative to YNG for the spatial task and the older ABT-263 group exhibited an increase in swim speed on the cue discrimination task. An increase in swim speed has been linked to anxiety, exposure to stress, and prior handling (Belviranli et al., 2012; Elmarzouki et al., 2014; Fridgeirsdottir et al., 2014; Rajab et al., 2014). Similarly, the latency to enter a novel environment is indicative of the level of anxiety (Chaouloff et al., 1997; Bell et al., 2009) and cross-sectional studies suggest that neophobia increases with age (Leite-Almeida et al., 2009; Febo et al., 2020). In this case, neophobia may increase more in males (Domonkos et al., 2017). Aged male rats, regardless of treatment, exhibited an increase latency to enter the dark compartment during the acquisition phase of inhibitory avoidance training (Budamagunta et al., 2023). For females, YNG and the ABT-263 treated group exhibited an increased latency to initially enter the dark compartment. The results suggest possible sex differences in anxiety or response to stress, which may have been exacerbated in ABT-263 treated females.

Sex differences in the effect of senolytic treatment were observed for the 24 h retention of inhibitory avoidance training. Aging males treated with vehicle exhibit an impaired memory, entering the dark compartment 24 h after receiving the shock and this memory impairment was not observed in the senolytic treated groups (Foster and Kumar, 2007; Budamagunta et al., 2023). In contrast, no memory impairment was observed for inhibitory avoidance in females, confirming previous finding of superior avoidance memory for females (Monleon et al., 2002; Foster et al., 2003; Colettis et al., 2022). Thus, sex differences in the effectiveness of senolytic treatment on memory for the inhibitory avoidance task are likely due to superior avoidance memory in aging females, relative to aging males.

An initial treatment effect was observed for the cue task, with the ADQ treated group exhibiting an increased latency and pathlength, possibly due to the stress of being placed in the watermaze, which may be enhanced in females (Perrot-Sinal et al., 1996; Beiko et al., 2004; Zorzo et al., 2023). The effect of swim stress can be reduced when the procedures or strategies for solving spatial tasks are acquired in youth (McQuail et al., 2020) or by employing cue discrimination as a pre-training procedure (Mabry et al., 1996; Foster, 2012; Guidi and Foster, 2012; McQuail et al., 2020). In fact, the latency and pathlength were reduced over the course of cue discrimination training in females and no group differences were observed for latency on subsequent testing on the spatial version of the watermaze task.

For the spatial version of the watermaze, young male 344 rats learn the location of the escape platform in a single day of training and remember the location over a 24 h period. Aged males also learn the 1-day watermaze task, observed as a decrease in distance to find the platform and, as a group, aged males perform above chance on the immediate probe trial and are not different from young rats (Foster, 2012). In the current study, the acquisition of a spatial discrimination strategy in females was similar to that observed for males and comparable our previous work in females (Foster et al., 2003; Bean et al., 2015). All female groups learned the spatial discrimination to about the same extent as indicated by the decrease in pathlength across training trials. Across all blocks, the ABT-263 treatment group exhibited an increased pathlength relative to YNG; however, no difference was observed for the final training blocks. Again, increased anxiety/stress of ABT-263 treated females may have resulted in an initial learning impairment, with all groups acquiring the task to about the same extent by the end of training. The conclusion that all groups exhibited a similar level of learning is supported by the acquisition probe trial search behavior, which was not different across groups for searching the goal and avoiding the opposite quadrant. Furthermore, search behavior was focused on the goal quadrant, such that the search time was above chance, across all groups, for the goal, and below chance for all groups, except AV, for the opposite quadrant. The results for the two studies indicate that regardless of treatment, following a single day of training, most aged male and female rats can acquire the spatial discrimination to a level comparable with young animals.

Young males retain the spatial discrimination memory over a 24 h retention period. However, starting in middle age, a subset of males exhibit forgetting, such that, as a group, the discrimination between the goal and opposite quadrant during the retention probe trial is not different from chance (Kumar and Foster, 2013; Guidi et al., 2014; Scheinert et al., 2015; Barter et al., 2021; Budamagunta et al., 2023). This age-related forgetting was ameliorated in males by the same senolytic treatment employed in the current study, such that the retention probe performance of older males treated with D + Q or ABT-263 was different from chance and not different from young males (Budamagunta et al., 2023). In the current study, only young females exhibited a search strategy focused on the goal quadrant during the retention probe trial, indicating that similar to males, young females maintained the spatial discrimination memory over the 24 h retention interval and retention is impaired in aging females. In contrast to results from senolytic treated aged male rats, forgetting was observed for older females treated with D + Q or ABT-263, such that search behavior was not different from chance during the 24 h probe trial for all older female groups. The results confirm that most aged male and female rats exhibit a decline in spatial episodic memory. However, in contrast to our work in male rats (Budamagunta et al., 2023), senolytic treatment did not improved memory in aging females.

The results indicate sex differences in senolytic effectiveness or responsiveness in maintaining cognition during aging. Beneficial treatment effects, limited to males, have been observed following injury induced cell senescence (Muralidharan et al., 2022; Schwab et al., 2022). For example, cellular senescence induced by traumatic brain injury was sensitive to senolytic treatment, decreasing senescent markers in males and increasing senescent markers in brains of female control animals (Schwab et al., 2022). Similarly, a differential response to senolytic treatment may disproportionately increase the survival of male mice infected with COVID-19 (Camell et al., 2021). The results of the current study add to this body of work, indicating that aging females are less responsive to senolytic treatment in preserving cognition and senolytic treatment may increase age-related sex differences in anxiety.

Dasatinib + quercetin reduced the age-related weight gain in males (Budamagunta et al., 2023). Since grip strength is normalized by weight, the weight loss may have contributed to an apparent increase in grip strength in older males. The senolytic treatments did not influence the age-related weight gain or grip strength in females suggesting a sex difference in weight gain following D + Q treatment may contribute to the absence of a treatment effect on grip strength for females. However, ABT-263 also increased grip strength in males, in the absence of a weight change. Furthermore, similar sex differences in age-related weight gain are observed for C57BL/6 mice (Mitchell et al., 2016) and D + Q and ABT-263 do not appear to consistently influence weight in a sexually dimorphic manner in C57BL/6 mice (Garbarino et al., 2020; Novais et al., 2021). Thus, the improvement in grip strength observed in males, and absent in females, is not likely due to differential effects of treatment on weight gain.

The differential benefits of senolytic treatment, favoring males, may be due to sex differences in the level of cellular senescence (Yousefzadeh et al., 2020). Sex hormones are thought to contribute to sex differences in aging processes, behavioral stress responsivity across the lifespan, and the effectiveness of interventions designed to delay aging or ameliorate age-related diseases (Bale and Epperson, 2015; Khaleghi et al., 2021; Knufinke et al., 2023; Polcz et al., 2023). As expected, our aging females underwent estropause, exhibiting an estrus cycle prior to treatment and no evidence of an estrus cycle at the end of the treatment period. Variability in cognitive decline is linked to sex hormones and previous work demonstrates that episodic memory on the watermaze declines in association with the decrease in estradiol (Warren and Juraska, 2000; Bean et al., 2014; Rentz et al., 2017). Estradiol may be protective against senescence-inducing stressors (Liu et al., 2016; Faubion et al., 2020; Ng and Hazrati, 2022), such that the increase in senescent markers and pro-inflammatory cytokines during menopause can be reduced by estradiol treatment (Gameiro and Romao, 2010; Faubion et al., 2020). However, if the observed memory deficit in aging females is due to increased cellular senescence associated with the loss of estradiol, then senolytic treatments should have preserved cognition in aging females. Alternatively, the mechanism for estradiol-mediated preservation of memory is thought to include rapid estradiol influences on hippocampal synaptic plasticity and the strength of synaptic connections, which are opposite to what is observed during aging (Foster, 2005). Impaired memory in males and females is linked to N-methyl-D-aspartate (NMDA) receptor hypofunction and alterations in synaptic plasticity processes that mediate episodic memory (Kumar and Foster, 2013; Kumar et al., 2018; Barter et al., 2020; Budamagunta et al., 2023). In males, senolytic treatment increases hippocampal synaptic transmission, increasing NMDA receptor function and episodic memory (Budamagunta et al., 2023, 2024). Similarly, activation of membrane estrogen receptors in females increases NMDA receptor function and episodic memory (Foster, 2005; Bean et al., 2015). Together, the results suggest that estradiol acts on additional aging mechanisms, independent of cellular senescence, such that the senolytic treatment may not have been able to compensate for the loss of estradiol.

## 5 Conclusion

The results of the current study suggest that senolytic treatment of females, starting in middle age, does not result in the same cognitive benefits observed in males. However, it is unclear if the treatments influenced the biology of aging in a similar manner in males and females. Thus, it will be important for future studies to examine sex differences in biological markers/mechanisms of senolytic treatments. A central question is whether there are sex differences in the ability of senolytic treatments to regulate brain mechanism of memory including regulation of NMDA receptor function.

## Data availability statement

The raw data supporting the conclusions of this article will be made available by the authors, without undue reservation.

## Ethics statement

The animal study was approved by Institutional Animal Care and Use Committee (IACUC) of University of Florida. The study was conducted in accordance with the local legislation and institutional requirements.

## Author contributions

AR: Data curation, Formal analysis, Investigation, Methodology, Writing – review & editing, Writing – original draft. LB: Data curation, Writing – review & editing. VB: Methodology, Writing – review & editing. AK: Data curation, Investigation, Methodology, Writing – review & editing. TF: Conceptualization, Data curation, Formal analysis, Funding acquisition, Project administration, Supervision, Writing – original draft, Writing – review & editing.
